# Probing locus coeruleus functional network in healthy aging and its association with Alzheimer’s disease biomarkers using pupillometry

**DOI:** 10.1186/s13195-025-01701-1

**Published:** 2025-02-27

**Authors:** Junjie Wu, Aaron Toporek, Qixiang Lin, Felicia C. Goldstein, David W. Loring, Michael A. Kelberman, David Weinshenker, Allan I. Levey, James J. Lah, Deqiang Qiu

**Affiliations:** 1 Department of Radiology and Imaging Sciences, School of Medicine, Emory University, 1364 Clifton Rd NE, Atlanta, GA 30322 USA; 2 Department of Neurology, School of Medicine, Emory University, Atlanta, GA USA; 3 School of Biological Sciences, Georgia Institute of Technology, Atlanta, GA USA; 4 Goizueta Alzheimer’s Disease Research Center, Emory University, Atlanta, GA USA; 5 Department of Human Genetics, School of Medicine, Emory University, Atlanta, GA USA; 6 Department of Molecular, Cellular, and Developmental Biology, University of Colorado Boulder, Boulder, CO USA; 7 Joint Department of Biomedical Engineering, Emory University and Georgia Institute of Technology, Atlanta, GA USA

**Keywords:** Locus coeruleus, Pupil diameter, Functional brain networks, MRI, Healthy aging, AD pathology

## Abstract

**Background:**

Alzheimer’s disease (AD) is the leading cause of dementia, and the early detection of the disease-associated changes allows early interventions. The locus coeruleus (LC) has been reported to be the first brain region to develop tau pathology in AD. However, the functional brain network of the LC in both healthy aging and AD pathology is largely unknown due to technical difficulties associated with the small size of the LC. In this study, we used the measurement of spontaneous pupil constriction/dilation as a surrogate for LC activity to study LC brain network changes during healthy aging.

**Methods:**

Thirty-seven healthy younger and thirty-nine healthy older adults were included from the Emory Healthy Brain Study and underwent resting-state functional MRI while simultaneously tracking pupil diameter. The measurements of pupil diameter dynamics were used as reference signals in brain connectivity analysis. The connectivity of the identified networks was then compared between younger and older participants. Correlations of the identified regions with neuropsychological assessments and cerebrospinal fluid (CSF) biomarkers were also evaluated.

**Results:**

A brain network of 20 clusters associated with pupil diameter dynamics was identified, including the LC as well as brain regions functionally connected to the LC. The pupil diameter network was found to positively correlate with the salience network and negatively correlate with the central executive network. Functional connectivity decreased within the pupil diameter network with healthy aging. The pupil diameter connectivity was associated with memory, executive, and visuospatial functioning. CSF total tau closely correlated with pupil diameter network.

**Conclusions:**

Pupil diameter dynamics provide valuable insights into LC-related processes. While they are not solely influenced by LC activity, spontaneous pupil constrictor/dilatory activity shows promise as a non-invasive approach to probe the LC network and warrants further studies to evaluate its value as an early biomarker of AD.

**Supplementary Information:**

The online version contains supplementary material available at 10.1186/s13195-025-01701-1.

## Background

Alzheimer’s disease (AD) is a neurodegenerative disorder affecting 10–30% of individuals aged 65 and older [1]. It is characterized by memory loss and cognitive decline [2], driven by amyloid beta (Aβ) plaques and tau neurofibrillary tangles [3–5], which contribute to synaptic loss and neuronal death [6–8]. Early detection is difficult even with recent advancements [1, 9], as cognitively normal (CN) elderly with AD pathology can remain asymptomatic for decades [10, 11]. Because of this, much research continues to focus on identifying biomarkers for improved risk assessment and early diagnosis.

The locus coeruleus (LC), a small region in the brainstem and the primary source of noradrenergic innervation [12, 13], is the first to develop tau pathology in AD [14–16], with hyperphosphorylated tau accumulating years before cognitive decline [14, 17–22]. Lower LC contrast in neuromelanin-sensitive MRI is associated with greater tau deposition, cognitive decline, and AD progression [23–28]. The LC’s critical role in AD has driven efforts to study its functional connectivity using functional MRI. Previous studies [29–32] used either atlas-based or individual subject region-of-interest (ROI) approaches to extract putative LC blood-oxygen level dependent (BOLD) time series for seed-based connectivity analysis. Comparatively, limited studies have investigated the effect of normal aging on the LC network [29, 31, 32] or its relationship with AD biomarkers [33]. Atlas-based analyses indicate that LC connectivity to the salience [31] and visual [29] networks decreases with age, while connectivity to the fronto-parietal cortex and the cerebellum [29] increases. Curvilinear connectivity patterns have also been observed with age [32]. Reduced LC activity and decreased connectivity with the hippocampus and parahippocampus are linked to faster cognitive decline, especially in the presence of elevated Aβ [33].

The location of the LC in the atlas-space is variable among subjects [34], rendering the atlas-based approaches potentially unreliable given the small size of the LC, which also makes it difficult to perform image segmentation and use it as a seed region due to a lower resolution typically achievable for functional MRI [35]. Despite these challenges, animal studies have demonstrated correlational and causal relationships between the LC activity and spontaneous pupil constriction/dilation [36–38]. Furthermore, pupillary responses have been shown to reflect LC dysfunction, suggesting their potential as a biomarker for early AD risk [39, 40]. Consequently, pupil diameter dynamics have been used as a non-invasive surrogate of the LC activity in studies examining the LC functional network [38, 41–44]. These studies indicate that the LC is functionally connected to the salience network comprised of the anterior cingulate cortex, insula, thalamus, and cerebellum. However, prior studies have focused solely on young adults, and no studies have explored age-related differences in pupil-based LC connectivity or its relationship with cerebrospinal fluid (CSF) biomarkers of AD and neuropsychological performance.

In the present study, we aimed to use pupil diameter as a surrogate for LC activity to characterize changes in the LC functional connectivity in the brain during healthy aging. To this end, simultaneous eye-tracking using a MR-compatible camera was performed during resting-state functional MRI (rsfMRI). The pupil diameter networks were compared between younger (< 57 years) and older (> 57 years) adults to identify connectivity differences associated with aging. Correlations of the network connectivity with neuropsychological performance and biomarkers of AD pathology, i.e., Aβ and tau in CSF, were estimated to understand the possible roles of the pupil diameter network in AD progression. We hypothesized that the connectivity within the pupil diameter network would differ between the younger and older groups. We further hypothesized that the network connectivity would be associated with memory, executive, and visuospatial functioning and with CSF phosphorylated tau.

## Methods

### Participants

Cognitively normal adults were recruited as part of the Emory Healthy Brain Study [45]. The initial participant pool consisted of 102 cognitively normal adults with complete rsfMRI data and complete eye-tracking data. These included 46 younger (age = 32.6 ± 12.1 years; 21 males) and 56 older (age = 65.9 ± 5.5 years; 15 males) participants, with older being defined as > 57 years [46]. This Health Insurance Portability and Accountability Act–compliant study was approved by the Emory University School of Medicine Institutional Review Board. Written informed consent was obtained prior to study participation from all participants in accordance with the Declaration of Helsinki.

### MRI and eye-tracking

MRI data were acquired on a Siemens Magnetom Prisma 3-Telsa scanner (Siemens Healthcare, Erlangen, Germany) equipped with a 32-channel head array coil. T1-weighted anatomical images were acquired using a magnetization-prepared rapid acquisition with gradient echo sequence (TR/TE = 2300/2.96 ms, TI = 900 ms, FA = 9°, voxel size = 1 × 1 × 1 mm^3^, 208 slices). A 10-min continuous BOLD imaging acquisition was performed using a multiband accelerated gradient-echo echo-planar imaging sequence (TR/TE = 1890/30 ms, FA = 52°, voxel size = 1.5 × 1.5 × 1.5 mm^3^, 81 slices, multiband factor = 3, 320 volumes). The participants were asked to focus on a fixed crosshair that can be seen through a mirror mounted on the head coil. At the same time as the rsfMRI, each participant’s right eye was tracked using an MRC Systems 12 M-i camera (focal length = 16 mm) and MRC Eye-Tracking software (MRC Systems, Heidelberg, Germany).

### CSF collection and analysis

Lumbar punctures were performed in the cognitively normal elderly using a 24-g Sprotte atraumatic spinal needle (Pajunk Medical Systems, Norcross, Georgia, USA) with aspiration. CSF was transferred from syringe to polypropylene tubes (Corning, Glendale, Arizona, USA) and frozen on dry ice within 1 h after collection. Aliquots (0.5 ml) were prepared from these samples after thawing (1 h) at room temperature and gentle mixing. Following a single freeze-thaw cycle, amyloid-β (Aβ) 1–42, total tau (T-tau), and tau phosphorylated at threonine 181 (P-tau) were measured using the multiplex xMAP Luminex platform (Luminex Corp., Austin, Texas, USA) with Innogenetics (INNO-BIA AlzBio3; Ghent, Belgium) immunoassay kit-based reagents. All assays were performed in a single laboratory (AKESOgen, Peachtree Corners, Georgia, USA). Subsequent aliquots were stored in bar code-labeled FluidX 0.9-ml polypropylene vials (Brooks Life Sciences, Chelmsford, Massachusetts, USA) at -80 °C. T-tau/Aβ ratio was calculated as an indicator of Aβ and tau burden.

### Neuropsychological assessments

A neuropsychological test battery was administered, including the Montreal Cognitive Assessment (MoCA) for overall cognitive status, the Free and Cued Selective Reminding Test (FCSRT) for verbal episodic memory, the Rey Complex Figure Test (RCFT) for visual memory and visuospatial functioning, the Judgment of Line Orientation (JoLO) for visuospatial ability, the Letter Fluency (FAS) for language and executive functioning, the Animal Fluency for language and semantic memory, the Trail Making Test Part A (TMTA) for processing speed, and the Trail Making Test Part B (TMTB) for executive functioning.

### Pupil diameter time series extraction

**Fig. 1:**
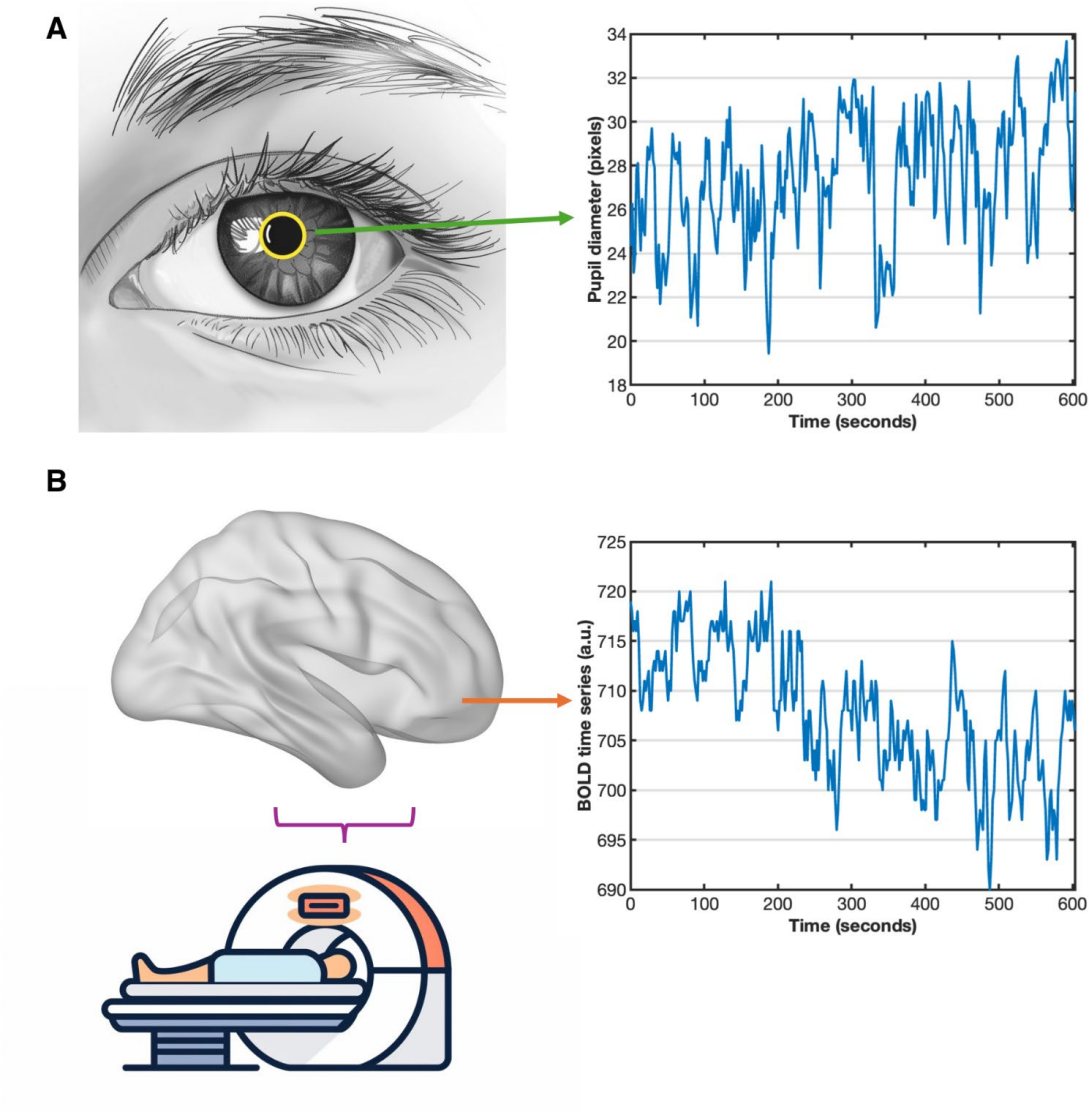
Schematic illustration of pupil diameter estimation and MRI data acquisition. (**A**) For HIPPA consideration, a synthetic eye-tracking photo was shown for illustration purposes with the pupil contour line (yellow) superimposed. The time course of the pupil diameter during resting-state functional MRI was obtained from the eye-tracking video. (**B**) After data acquisition, the functional MRI blood-oxygen level dependent (BOLD) time series was extracted from each voxel in the brain

The raw eye-tracking videos were processed using DeepVOG, an open-source tool that uses a neural network to segment the pupil in each frame of the eye-tracking video [47]. See Fig. 1A for an illustration of pupil segmentation and pupil diameter dynamics. As a quality control procedure, participants were included for further analyses if less than 20% of their eye-tracking frames were unsuccessful and at least 75% of their eye-tracking frames had a confidence level > 0.9, as determined by DeepVOG. These criteria were chosen to balance data quality and inclusion of data points, ensuring reliable connectivity results while minimizing the exclusion of participants.

After acquiring the raw pupil diameter data, the data was further processed to obtain a pupil diameter time series, with one timepoint per MRI volume acquired. First, all frames with a confidence level < 0.9 were filtered out by flagging them as missing values. Then, the data was smoothed using a sliding window filter with a window size of 57 frames and convolved with the canonical hemodynamic response function (HRF). Finally, the pupil diameter at each timepoint corresponding to when an MRI volume was acquired was obtained using linear interpolation. Additionally, the first derivative of the resultant pupil diameter data was also calculated. We will use the terms “pupil diameter time series” and “first derivative time series” to refer to the two time series after HRF convolution respectively. Only these two HRF convolved time series were used for further analyses.

### MRI preprocessing

rsfMRI data (illustrated in Fig. 1B) were preprocessed using CONN toolbox [48] (https://www.conn-toolbox.org). First, within each participant, rsfMRI volumes were slice-timing corrected, and every volume was realigned to the first acquired volume. Next, each participant’s functional data was co-registered to their T1-weighted structural image, and then both the functional and structural data were normalized to Montreal Neurological Institute (MNI) space. An 8 mm full-width-at-half-maximum Gaussian kernel was then applied to smooth the rsfMRI images. This level of smoothing helps mitigate inter-subject variability, and facilitate the detection of large-scale functional networks without excessively blurring anatomical details. Outlier volumes for each participant were identified using CONN’s scrubbing tool with intermediate settings, which defined an outlier as a volume with a framewise displacement greater than 0.9 mm or a global BOLD signal change above 5 standard deviations [49, 50]. Finally, denoising was performed using Ordinary Least Squares regression in CONN’s default denoising pipeline to remove the effects of confounding variables such as motion, rest, white matter signal, CSF signal, and outlier volumes, and a bandpass filter was applied to remove all signal outside of the frequency band between 0.008 and 0.09 Hz [49, 51]. The global BOLD signal was not regressed out because its removal could potentially introduce artificial negative correlations in functional network analysis [52], and it would not effectively reduce non-neuronal noise sources related to the external pupil diameter dynamics. The effect of rest was removed to eliminate transient ramping effects at the beginning of each rsfMRI acquisition.

### Functional network analysis

The pupil diameter and their first derivative time series were used as the seed time series respectively in two separate seed-to-voxel analyses. Pearson correlation coefficients were computed between the seed time series and the signal in each target voxel throughout the brain. The resulting correlation coefficients were then Fisher z-transformed. Also, voxel-wise local correlation, a measure of regional homogeneity, was calculated to reflect the local functional coherence of a given voxel with its neighboring voxels [53].

Brain clusters co-activated with either time series were identified using one-sample t-test across all participants. Group differences were examined to identify brain clusters differentially correlated with either time series between younger and older participants using general linear models with sex as a covariate. Candidate clusters were identified using an uncorrected voxel-level *P*-value threshold of 0.001. Each cluster’s size was then compared to a distribution of known sizes to generate a cluster-level *P*-value. Clusters with a false discovery rate (FDR) corrected cluster-level *P*-value less than 0.05 were deemed significant.

### Correlation analysis with neuropsychological and CSF assessments

For each cluster identified from the pupil diameter or first derivative analysis, associations between functional connectivity and neuropsychological performance or CSF biomarker measurements were evaluated using multiple regression with age and sex as covariates. FDR correction was applied for multiple comparisons. The analyses were repeated for local correlation to examine whether the observed associations with pupil diameter connectivity were driven by local brain activity or LC-mediated connectivity.

## Results

### Dataset

The pupil time series selection criteria, designed to balance data quality and inclusion of data points, resulted in a dataset of 76 cognitively normal adults (Table 1). Of these, 37 participants were younger (age = 30.6 ± 10.6 years; 15 males) and 39 were older (age = 66.0 ± 5.7 years; 7 males).

**Table 1:** Demographics, CSF, and neuropsychological assessments

	Groups		Older vs. Younger^1^	
	Younger	Older	*t*-statistic	*P*-value
*N*	37	39		
Age (years)	30.6 ± 10.6	66.0 ± 5.7	18.248	< 0.001^***^
Sex (male)	15	7		0.051
Education (years)	16.8 ± 1.3	16.4 ± 2.0	-1.048	0.372
Aβ (pg/dl)	NA	535.5 ± 145.2	NA	
T-tau (pg/dl)	NA	58.6 ± 28.9	NA	
P-tau (pg/dl)	NA	29.7 ± 13.9	NA	
T-tau/Aβ ratio	NA	0.128 ± 0.092	NA	
MoCA	28.6 ± 1.3	26.4 ± 2.6	-3.114	0.020^*^
FCSRT free recall	40.4 ± 4.6	35.9 ± 3.4	-2.928	0.025^*^
RCFT immediate free recall	26.2 ± 3.7	19.1 ± 7.3	-2.556	0.034^*^
RCFT delayed free recall	25.3 ± 4.0	16.8 ± 8.3	-2.838	0.025^*^
Recognition of RCFT elements	21.8 ± 1.0	19.9 ± 1.7	-3.353	0.016^*^
RCFT copy accuracy score	33.2 ± 3.2	32.2 ± 2.9	-0.378	0.759
JoLO	25.4 ± 2.9	24.2 ± 4.2	-0.187	0.853
Letter Fluency (FAS)	45.9 ± 7.8	43.2 ± 10.7	-0.601	0.637
Animal Fluency	24.4 ± 4.4	22.8 ± 3.8	-1.306	0.275
TMTA	25.6 ± 9.6	35.8 ± 13.4	2.270	0.051
TMTB	52.4 ± 11.2	76.4 ± 30.7	2.693	0.029^*^
TMTB - TMTA	26.8 ± 11.1	40.6 ± 22.5	2.089	0.068

Aβ, Amyloid-β 1–42; T-tau, total tau; P-tau, tau phosphorylated at threonine 181; MoCA, Montreal Cognitive Assessment; FCSRT, Free and Cued Selective Reminding Test; RCFT, Rey Complex Figure Test; JoLO, Judgment of Line Orientation; TMTA, Trail Making Test Part A; TMTB, Trail Making Test Part B

^1^Group differences between older (> 57 years) and younger (< 57 years) adults were evaluated using an independent *t*-test (for age and education), a chi-square test (for sex), general linear models with sex as a covariate (for cerebrospinal fluid and neuropsychological assessments)

Significant at ^*^P < 0.05 and ^***^P < 0.001, false discovery rate corrected

Comparison of neuropsychological assessments (Table 1) showed the healthy older participants performed significantly worse than the healthy younger participants on the MoCA (*P* = 0.020, FDR corrected), FCSRT free recall (*P* = 0.025, FDR corrected), RCFT immediate free recall (*P* = 0.034, FDR corrected), RCFT delayed free recall (*P* = 0.025, FDR corrected), recognition of RCFT elements (*P* = 0.016, FDR corrected), and TMTB (*P* = 0.029, FDR corrected).

### Seed-to-voxel analysis using pupil diameter time series

**Fig. 2:**
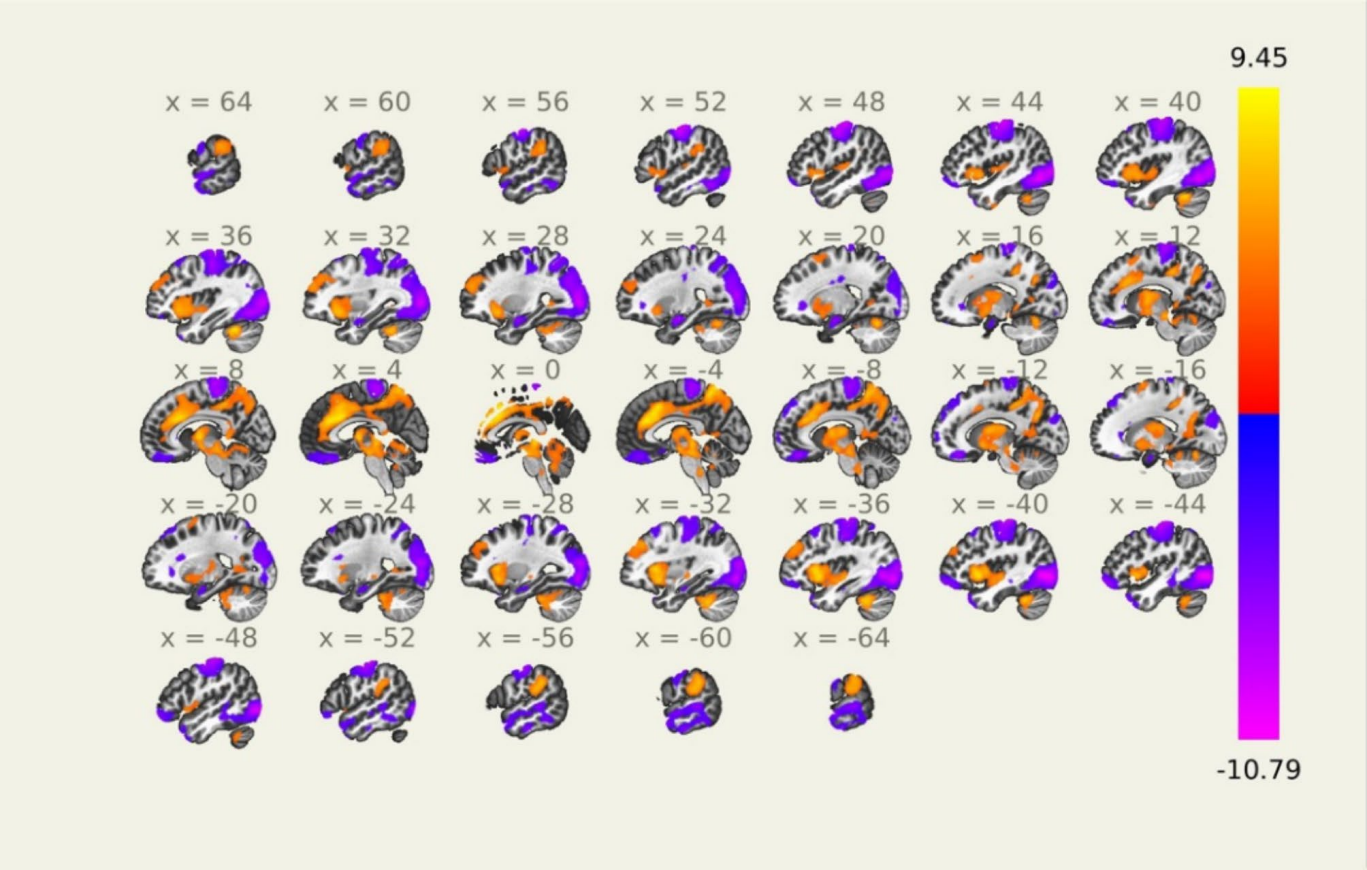
Seed-to-voxel analysis using the pupil diameter time series as a seed signal. Voxels positively correlated across all participants are shown in red, while those negatively correlated are shown in blue. No significant differences were found when comparing younger (< 57 years) and older (> 57 years) participants

The clusters identified using the pupil diameter time series as a seed signal are shown in Fig. 2. A total of 23 brain regions/voxel clusters were identified as significantly correlated with the pupil diameter time series across all participants. Three clusters identified were found to be unlabeled or in white matter, and thus were removed from further analysis. Of the remaining 20 clusters, 8 positively correlated with the pupil diameter time series, and 12 negatively correlated (Fig. 2 and Supplementary Table S1). The largest cluster was positively correlated and consisted of over 20,000 voxels. Its peak voxel was in the anterior cingulate, and the cluster also included voxels in the precuneus, cerebellum, and brainstem. Notably, the LC overlapped with the voxels in the largest cluster. Central executive, visual, and sensorimotor regions, as well as parts of the limbic system, were found to be negatively correlated with the pupil diameter time series. Regions belonging to the salience network, including the anterior cingulate and the supramarginal gyrus, were found to be positively correlated.

When comparing younger (< 57 years) and older (> 57 years) groups, no brain regions were found to be differentially correlated with pupil diameter time series.

### Seed-to-voxel analysis using first derivative time series

**Fig. 3:**
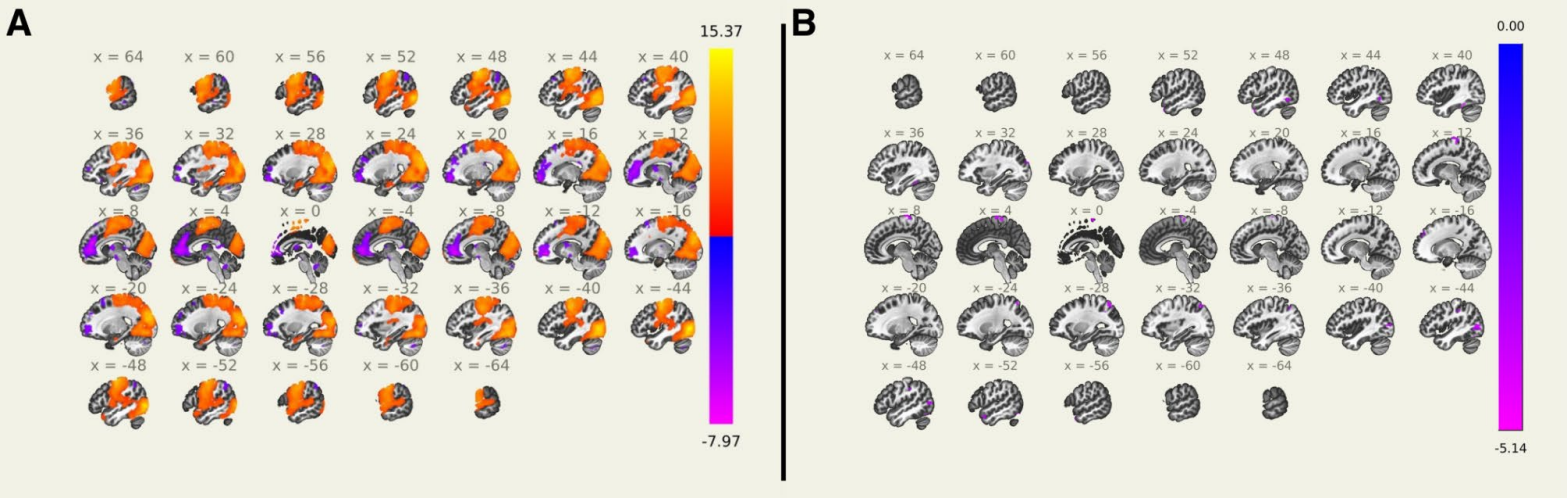
Seed-to-voxel analysis using the first derivative time series as a seed signal. (**A**) Voxels positively correlated across all participants are shown in red, while those negatively correlated are shown in blue. (**B**) Voxels more correlated in older participants (> 57 years) are shown in red, while those more correlated in younger participants (< 57 years) are shown in blue. All clusters identified were less correlated with the first derivative of pupil diameter in older participants (> 57 years)

The clusters identified using the first derivative time series as a seed signal are shown in Fig. 3. A total of 21 clusters were identified as significantly correlated with the first derivative time series across all participants. One cluster was found to be unlabeled and in the white matter, and thus was removed from further analysis. Of the remaining 20 clusters, 4 positively correlated with the first derivative time series, and 16 negatively correlated (Fig. 3A and Supplementary Table S2). The LC did not overlap with any of the identified clusters. The largest cluster was positively correlated and consisted of nearly 66,000 voxels covering large portions of the occipital, temporal, and parietal lobes, with its peak voxel located in the lateral occipital cortex. Several medial frontal regions were found to be negatively correlated.

Eleven clusters were found to have significantly lowered correlation with the first derivative time series in the older participants (> 57 years) compared to the younger participants (< 57 years) (Fig. 3B and Supplementary Table S3). These included the medial temporal regions near the fusiform gyrus, temporal poles, parts of the lateral occipital cortex, and the precentral gyrus.

### Correlations with neuropsychological performance

Table 2 presents the correlations of functional connectivity with neuropsychological assessments using data from both the younger and older groups. Eleven clusters were identified as significantly correlated with neuropsychological assessments (FDR corrected), all of which involved tests evaluating memory, executive, or visuospatial functioning. Notably, the anterior supramarginal gyrus and superior frontal gyrus were correlated with FCSRT free recall. The results remained consistent when education was included as an additional covariate in the multiple regression analysis alongside age and sex (Supplementary Table S4). Among older participants, all significant correlations with functional connectivity were related to memory, executive, or visuospatial functioning (Supplementary Table S5), consistent with the results from the full sample. None of the cluster-neuropsychological performance correlations were significant for local correlation (Supplementary Table S6), suggesting that the associations between pupil diameter connectivity and neuropsychological performance were driven by LC-mediated connectivity rather than local brain activity.

**Table 2:** Correlations of functional networks with neuropsychological performance

Cluster name	Peak coordinates in MNI space	Neuropsychological assessment	Test domain	*r*-statistic	*P*-value	
Pupil diameter time series as a seed signal						
Anterior supramarginal gyrus left	-58 -38 + 32	FCSRT free recall	Memory	0.580	< 0.001	
Anterior supramarginal gyrus right	+ 68 − 30 + 30	FCSRT free recall	Memory	0.448	0.022	
Superior frontal gyrus left	-16 + 4 + 70	FCSRT free recall	Memory	0.467	0.022	
unlabeled*	+ 20 + 32 − 30	FCSRT free recall	Memory	-0.488	0.016	
Frontal pole right	+ 46 + 48 − 16	Letter Fluency (FAS)	Language and executive	0.456	0.022	
Middle frontal gyrus left	-30 + 36 + 38	TMTA	Executive	0.419	0.044	
Hippocampus left	-22 -10 -18	TMTB– TMTA	Executive	0.451	0.022	
First derivative time series as a seed signal						
Posterior middle temporal gyrus right	+ 60 − 26 -12	RCFT immediate free recall	Memory	-0.513	0.006	
Vermis 9	+ 0–50 -36	RCFT delayed free recall	Memory	-0.459	0.022	
Hippocampus right	+ 32 − 8 -22	RCFT copy accuracy score	Visuospatial	0.574	< 0.001	
Precuneus*	+ 0–62 + 64	JoLO	Visuospatial	0.497	0.008	

MNI, Montreal Neurological Institute; FCSRT, Free and Cued Selective Reminding Test; TMTA, Trail Making Test Part A; TMTB, Trail Making Test Part B; RCFT, Rey Complex Figure Test; JoLO, Judgment of Line Orientation

The associations were evaluated using multiple regression with age and sex as covariates. *P*-values were adjusted for multiple comparisons using false discovery rate correction

Clusters were named based on the CONN atlas regions of the peak voxels. If the peak voxel was not labeled, the immediately adjacent 26 voxels were scanned, and the cluster was named based on the adjacent voxels’ labels, with an asterisk (*) appended to the end of the cluster name. If neither the peak voxel nor the immediately adjacent voxels were labeled in the CONN atlas, the cluster was named as “unlabeled*”

### Correlations with CSF biomarkers

Table 3 shows the correlations between functional connectivity and CSF biomarkers using data from the elderly group. Two clusters were identified as being positively associated with CSF biomarker measurements (FDR corrected). Of the 3 significant cluster-CSF biomarker correlations, two involved the frontal poles and total tau levels. None of the cluster-CSF biomarker correlations reached significance for local correlation (Supplementary Table S7), indicating that the relationships between pupil diameter connectivity and CSF biomarkers were driven by LC-mediated connectivity rather than local brain activity.

**Table 3:** Correlations of functional networks with CSF biomarkers

Cluster name	Peak coordinates in MNI space	CSF biomarker	*r*-statistic	*P*-value	
Pupil diameter time series as a seed signal					
Frontal pole left	-10 + 64 + 0	T-tau	0.477	0.042	
Frontal pole left	-10 + 64 + 0	T-tau/Aβ ratio	0.501	0.042	
First derivative time series as a seed signal					
Frontal pole right	+ 4 + 62 − 26	T-tau	0.554	0.008	

MNI, Montreal Neurological Institute; CSF, cerebrospinal fluid; T-tau, total tau; Aβ, Amyloid-β 1–42

The associations were evaluated using multiple regression with age and sex as covariates. *P*-values were adjusted for multiple comparisons using false discovery rate correction

Clusters were named based on the CONN atlas regions of the peak voxels

## Discussion

We used pupil diameter as a surrogate of LC activity to study the LC network. Brain networks of 20 clusters were identified to correlate with pupil diameter and its first derivative respectively at rest in healthy younger (< 57 years) and older (> 57 years) participants. Brain activities in the sensorimotor and visual regions were found to negatively correlate with pupil diameter changes (first derivative), in agreement with previous studies [38, 42]. More importantly, regions of the midbrain and brainstem, including the LC, were found to positively correlate with pupil diameter, also in agreement with previous studies [38, 42]. Our study extended these previous studies by investigating the difference in the pupil dynamics network between younger (< 57 years) and older (> 57 years) adults as well as the relationship of pupil dynamics network with CSF biomarkers of AD and neuropsychological performance.

A cluster, including the brainstem and the LC, was found to be functionally associated with spontaneous pupil diameter dynamics. Moreover, similar regions to the ones we identified in the present study have been reported to be functionally connected to the LC in young adults, including the anterior cingulate and cerebellum [29, 30]. Studies in mice and rhesus macaques have found pupil dilation to be directly correlated with an increase in LC activity and norepinephrine turnover [36, 37]. Furthermore, selective chemogenetic or optogenetic activation of the LC rapidly elicits pupil dilation in mice and rats, indicating a causal effect [37, 54–56]. Additionally, a study in humans found pupil dilation to be indirectly associated with LC activation through an increase in BOLD activity in a region of the brain that included the LC [38]. Consistent with these previous studies, our findings lend additional support to the use of pupil diameter as a proxy for LC activity.

Several regions comprising the salience network, including the supramarginal gyrus and the anterior cingulate, were found to be positively correlated with pupil constrictor/dilatory activity in our analysis, suggesting strong connectivity between the salience network and the LC. In contrast, regions of the frontoparietal network, or central executive network, were found to be negatively correlated with pupil diameter. The salience network is thought to function as a center for the integration of sensory information and modulates the switching between internally and externally directed cognition, which are controlled by the default mode and central executive networks, respectively [57]. The positive correlation with pupil diameter indicates that spontaneous changes in pupil diameter may be reflective of task-independent changes in arousal state which could be modulated in part by the LC [42]. This is further supported by the negative correlation between pupil diameter and the central executive network, and the lack of a correlation between pupil diameter and regions of the default mode network. Because the patients were at rest during the scan, there was less externally directed cognition, leading to the deactivation within the central executive network. On the other hand, the lack of activation within the default mode network does not necessarily indicate no internal cognition. Instead, pupil diameter is more indicative of the modulation of arousal state rather than the state itself. However, no regions were found to be differentially correlated with pupil diameter when comparing between younger and older participants. This could be simply because all participants were cognitively normal in our study. It was previously reported that LC connectivity to the salience network decreases in cognitively normal older adults compared to younger adults [31]. However, the LC is a very small brain region with an extent of about 2 mm in the axial plane. The use of a population LC template mask to extract the LC BOLD time series is not validated and is potentially problematic since even a minor mis-registration could result in a large error due to the small size of the LC in the axial plane. Further technical development of high-resolution imaging methods such as those at 7T might provide additional tools for additional insights, and is currently being pursued by us.

Previous studies utilizing rsfMRI in humans [43] and electrophysiological recordings in mice [58] suggest that the rate of pupil diameter change, rather than pupil diameter itself, may better reflect LC activity. This aligns with the role of the LC-noradrenergic system in phasic arousal, which is thought to drive transient pupil fluctuations [58, 59]. To investigate this, we identified a brain network associated with the first derivative of pupil diameter at rest across all participants. Consistent with a previous study [43], we found distinct patterns of spatial correlation for the neural correlates of pupil size and its first derivative. These findings suggest different neural dynamics underlying sustained pupil states and rapid pupil changes, with the pupil diameter indicating tonic arousal, while its first derivative represents transient responses [59]. Interestingly, unlike with pupil diameter, brain networks co-activated with the first derivative of pupil diameter dynamics did not include the LC regions, implying that LC activity at rest may be more closely linked to tonic arousal than to transient pupil fluctuations. A large visual region was found to be positively correlated with the first derivative, while a medial frontal region was negatively correlated. Both these findings are inconsistent with the previous study [43]. However, the insular areas of the salience network were positively correlated with the first derivative, in agreement with the previous study [43]. These discrepancies could be due to the selective inclusion of young participants less than 35 years old in the previous study [43]. They could also be due to the previous study’s greater vigilance in keeping participants awake during the entire duration of the scan, as visual and sensorimotor regions are known to be positively correlated with drowsiness, while the salience network helps regulate tonic alertness [43, 60, 61]. In the current study, patients were not repeatedly asked to open their eyes, and some fell asleep during the scan. While all these participants were excluded from our final analysis in our eye-tracking quality control, the positive correlation of the first derivative with the visual region could be due to increased drowsiness of the participants who were analyzed. At the same time, the positive correlation with the insular regions of the salience network could be a result of changes in tonic alertness owing to drowsiness.

Eleven small clusters were identified to be significantly less correlated to the first derivative of pupil diameter in older participants than in younger participants. These included medial temporal regions near the fusiform gyrus, which is known to play a role in facial recognition [62]. The temporal lobe, including the fusiform gyrus, is one of the first cortical regions to develop neurofibrillary tangle pathology in AD progression [63]. Tau pathology is hypothesized to spread via synaptic connections from the LC and other brainstem nuclei directly to the entorhinal cortex, and then to the rest of the temporal lobe [14, 15]. Alterations in the functional connectivity of the fusiform gyrus with other brain regions in MCI is hypothesized to cause decreased visual cognition [62]. While the LC was not implicated by Cai et al. as a region with altered connectivity to the fusiform gyrus [62], Lee et al. showed that connectivity between the LC and the fusiform gyrus is higher in older adults compared to younger adults, and hypothesized that this could contribute to reduced attentional control [31].

The 11 significant cluster-neuropsychological score correlations involved selective reminding, complex figure testing, trail-making, judgement of line orientation, or letter fluency. This indicates that the pupil diameter network includes brain regions involved in memory, executive functioning, and visuospatial ability. This also supports the hypothesis that the LC is functionally connected to these regions to some degree, given the LC’s role in the modulation of cognition [64, 65]. In fact, the proper functioning of the LC is proposed to promote working memory capacity through modulation of the frontoparietal network [66]. In this model, the salience network reciprocally regulates the LC, which in turn modulates the frontoparietal network, which then normally suppresses the default mode network, allowing for high working memory capacity during external cognition [66]. However, if the LC is impaired, the frontoparietal network fails to sufficiently suppress the default mode network, which can lead to deficits in working memory [66]. This dysregulation in attention control has been implicated in several diseases, including AD [67]. Our finding further corroborates the role of LC-salience network crosstalk in cognition regulation, and the fact that LC degeneration could lead to profound cognitive deficits.

Among the 3 significant brain cluster-CSF biomarker correlations, two involved the frontal poles and total tau. CSF total tau is believed to reflect neuronal damage [68], instead of the burden of tau tangles in the brain. The underlying mechanisms of the associations between pupil diameter connectivity and total tau are not clear. They could be explained by subtle cerebrovascular damage represented by white matter hyperintensities on MRI, which is prevalent in older age. Such MRI marker is correlated with CSF total tau [69], and a recent study suggested that vascular cognitive dysfunction was associated with aberrant pupillary responses, more pronounced than those seen in AD [70]. On the other hand, damage to the LC was found to exacerbate tau pathology in the forebrain [71], potentially leading to neuronal damage and elevated CSF total tau levels [68, 72, 73]. The observed positive correlations might indicate a compensatory response to early neuronal damage in asymptomatic AD. Interestingly, phosphorated tau in the CSF did not show any correlation with the LC connectivity in this study. Moreover, the cluster encompassing the brainstem and LC was not associated with either total tau or phosphorated tau in our analysis, despite tau accumulation in these regions during normal aging and the earliest stages of AD [18, 19, 22, 74, 75]. Notably, while CSF P-tau 181 measured in the present study was previously thought to represent tau deposition in the brain, a recent study challenged this belief and showed that CSF P-tau 181 correlates more strongly with amyloid PET than with tau PET [76]. Studies using tau PET are needed to further explore the relationship between pupil diameter connectivity and tau.

Pupil diameter is not solely reflective of LC activity. While pupil size is generally linked to LC spiking, this relationship exhibits significant variability and is influenced by behavioral and cognitive states, including arousal, attention, and decision bias [77]. This variability suggests that pupil dilation is influenced by a number of brain regions besides the LC [78], including the hypothalamus, which projects brain-wide [79] and was also found to correlate with pupil diameter and its first derivative in our study. Furthermore, other neural structures including the dorsal raphe nucleus [80], the pretectal olivary nucleus [81], and the superior colliculus [81], could potentially contribute to the functional associations between the brainstem and pupil diameter dynamics. Many of these regions have functional connections with the LC and each other. For example, orexin neurons in the hypothalamus can also drive pupil dilation [79]. These effects of orexin appear to be transmitted through and require the LC [79], but that may not be true of the other neuroanatomical substrates. All these regions can have confounding effects on the identified network activity and connectivity, such that it is not exclusively attributed to the LC. Therefore, the results of this study should be interpreted cautiously about the LC. Future studies utilizing higher spatial resolution at ultra-high field MR imaging may help overcome current technical difficulties in the ROI-based approaches. This could provide more accurate estimates of the LC functional connectivity and clarify the contributions of different nuclei to spontaneous pupil dilation/constriction during rest. Moreover, comparisons between the pupil diameter network and the LC functional connectivity are required in future research to enhance our understanding of these relationships.

Several issues need to be addressed in the future. First, this is a cross-sectional study, which means that some of the differences (or lack thereof) observed between younger and older groups could be due to individual differences due to sampling biases. A longitudinal study would circumvent these differences by allowing for the comparison of the pupil brain networks in the same participants over time. Second, a higher proportion of female participants, particularly in the older age group, were recruited in this study, likely due to women’s longer life expectancy and greater willingness to participate in research. This resulted in findings that are skewed toward females. Future studies should aim to achieve a more balanced sample distribution between males and females to ensure broader generalizability of the results. Third, there is a lack of robust pupil segmentation. Despite using a machine-learning based approach, the segmentation tool still struggled to properly segment dark-colored eyes. This resulted in a significant portion of participants being discarded from the analysis. Improvement of current pupil segmentation tools to better identify the pupil in such cases would allow a much larger number of participants to be included in the final analysis, thereby increasing the statistical power of the analysis. Finally, this study did not collect medication use information for all participants, which could influence norepinephrine signaling, LC connectivity, or arousal. Although approximately half of the participants were relatively young and less likely to be on such medications, this potential confounder cannot be ruled out. Future studies should account for medication use.

## Conclusions

In conclusion, two separate brain networks associated with pupil diameter and its first derivative were identified in cognitively normal participants using resting-state functional MRI. The pupil diameter network positively correlated with the salience network and negatively correlated with the central executive network. Functional connectivity decreased within pupil diameter network with healthy aging. Moreover, pupil diameter network was associated with memory, executive, and visuospatial functioning. Total tau closely correlated with pupil diameter connectivity. These findings suggest that pupil diameter is a reasonable surrogate of the LC activity, and could be used to track changes in functional connectivity in aging, and possibly in AD progression.

## Electronic supplementary material

Below is the link to the electronic supplementary material.

Supplementary Material 1

## Data Availability

Due to privacy considerations, the original eye-tracking and brain image data are not available for sharing. The derived data that support the findings of this study are available upon reasonable request from qualified investigators, adhering to ethical guidelines and signing a data use agreement with the authors’ institution.

## Abbreviations

AD: Alzheimer’s disease
Aβ: Amyloid beta
CN: Cognitively normal
LC: Locus coeruleus
ROI: Region-of-interest
BOLD: Blood-oxygen level dependent
CSF: Cerebrospinal fluid
rsfMRI: Resting-state functional MRI
T-tau: Total tau
P-tau: Tau phosphorylated at threonine 181
MoCA: Montreal Cognitive Assessment
FCSRT: Free and Cued Selective Reminding Test
RCFT: Rey Complex Figure Test
JoLO: Judgment of Line Orientation
TMTA: Trail Making Test Part A
TMTB: Trail Making Test Part B
HRF: Hemodynamic response function
MNI: Montreal Neurological Institute
FDR: False discovery rate
