# Supplementary material for: Probing locus coeruleus functional network in healthy aging and its association with Alzheimer’s disease biomarkers using pupillometry
Source: Alzheimers Res Ther. 2025 Feb 27;17:53. doi: 10.1186/s13195-025-01701-1 (PMC11866666; doi:10.1186/s13195-025-01701-1)
Supplement: Supplementary file 1 — Supplementary Material 1 [file 13195_2025_1701_MOESM1_ESM.pdf]

Probing locus coeruleus functional network in healthy aging and its association with Alzheimer's disease biomarkers using pupillometry

Supplementary Table S1. Voxel clusters significantly correlated with the pupil diameter time series

Cluster name	Peak coordinates in MNI space	Cluster size (voxels)
Anterior cingulate	+0 +32 +24	20410
Inferior lateral occipital cortex right	+42 -66 -8	12096
Inferior lateral occipital cortex left	-40 -80 -4	8634
Postcentral gyrus left	-42 -26 +68	2807
Medial frontal cortex*	-8 +40 -20	1509
Temporal pole right	+40 +20 -40	1202
Anterior supramarginal gyrus left	-58 -38 +32	1024
Anterior supramarginal gyrus right	+68 -30 +30	840
unlabeled*	-18 +38 +56	831
Frontal pole left	-44 +46 -14	757
Frontal pole right	+30 +52 +26	732
Middle frontal gyrus left	-30 +36 +38	639
Amygdala right	+24 -8 -18	389
Frontal pole right	+46 +48 -16	341
Hippocampus left	-22 -10 -18	251
**unlabeled***	**+20 +32 +0**	**171**
Superior frontal gyrus left	-16 +4 +70	156
unlabeled*	+46 -10 -48	119
Middle frontal gyrus right*	+38 +26 +56	118
**unlabeled***	**-22 +22 +20**	**114**
**unlabeled***	**-20 +30 +0**	**97**
Frontal pole left	-10 +64 +0	86
unlabeled*	+20 +32 -30	74

MNI, Montreal Neurological Institute.

Clusters shown in red positively correlated with pupil diameter, while clusters shown in blue negatively correlated.

Clusters were named based on the CONN atlas regions of the peak voxels. If the peak voxel was not labeled, the immediately adjacent 26 voxels were scanned, and the cluster was named based on the adjacent voxels' labels, with an asterisk (*) appended to the end of the cluster name. If neither the peak voxel nor the immediately adjacent voxels were labeled in the CONN atlas, the cluster was named as "unlabeled*". If a cluster's peak voxel was unlabeled and the cluster was clearly located in the white matter, the cluster was subsequently discarded from further analysis (bolded).

Supplementary Table S2. Voxel clusters significantly correlated with the first derivative time series

Cluster name	Peak coordinates in MNI space	Cluster size (voxels)
Inferior lateral occipital cortex left	-46 -76 +0	65931
Paracingulate gyrus left	-10 +44 -4	6299
unlabeled*	+2 -40 +8	937
Hippocampus right	+32 -8 -22	516
Hippocampus left	-26 -6 -26	387
Frontal pole right	+4 +62 -26	377
Angular gyrus right	+48 -50 +42	359
Cerebellum crus2 left	-38 -70 -38	297
Cerebellum crus1 right	+36 -56 -30	293
unlabeled*	+10 -10 -12	271
Superior frontal gyrus right	+18 +24 +62	267
Angular gyrus left	-52 -52 +40	219
Superior frontal gyrus left	-16 +18 +66	218
Vermis 9	+0 -50 -36	157
**unlabeled***	**+14 -34 +24**	**131**
Frontal pole left	-22 +46 +32	96
Frontal orbital cortex right	+32 +22 -14	90
Middle frontal gyrus left	-26 +26 +34	79
Precuneus*	+0 -62 +64	71
Posterior middle temporal gyrus right	+60 -26 -12	67
Middle frontal gyrus right	+42 +22 +40	64

MNI, Montreal Neurological Institute.

Clusters shown in red positively correlated with the first derivative of pupil diameter, while clusters shown in blue negatively correlated.

Clusters were named based on the CONN atlas regions of the peak voxels. If the peak voxel was not labeled, the immediately adjacent 26 voxels were scanned, and the cluster was named based on the adjacent voxels' labels, with an asterisk (*) appended to the end of the cluster name. If neither the peak voxel nor the immediately adjacent voxels were labeled in the CONN atlas, the cluster was

named as “unlabeled*”. If a cluster’s peak voxel was unlabeled and the cluster was clearly located in the white matter, the cluster was subsequently discarded from further analysis (bolded).

Supplementary Table S3. Voxel clusters differentially correlated with the first derivative time series between young and older participants

Cluster name	Peak coordinates in MNI space	Cluster size (voxels)
Precentral gyrus right	+08 -20 +78	352
Lateral occipital cortex, inferior division left	-44 -70 +06	212
Lateral occipital cortex, superior division left	-28 -66 +52	182
Inferior temporal gyrus, temporooccipital part right	+46 -56 -10	77
Temporal fusiform cortex, posterior division right	+40 -36 -26	72
Temporal pole left	-52 +08 -24	60
Frontal pole left	-14 +50 +32	56
Lateral occipital cortex, superior division right	+32 -86 +24	53
Temporal pole right	+48 +18 -34	53
Supramarginal gyrus, anterior division left	-46 -32 +38	48
Lateral occipital cortex, inferior division left	-52 -64 -16	48

MNI, Montreal Neurological Institute.
All clusters identified were less correlated with the first derivative of pupil diameter in older participants.
Clusters were named based on the CONN atlas regions of the peak voxels. If the peak voxel was not labeled, the immediately adjacent 26 voxels were scanned, and the cluster was named based on the adjacent voxels' labels, with an asterisk (*) appended to the end of the cluster name.

Supplementary Table S4. Correlations of functional networks with neuropsychological performance using multiple regression controlling for age, sex, and education

Cluster name	Peak coordinates in MNI space	Neuropsychological assessment	Test domain	*r*-statistic	*P*-value
Pupil diameter time series as a seed signal					
Anterior supramarginal gyrus left	-58 -38 +32	FCSRT free recall	Memory	0.640	< 0.001
Anterior supramarginal gyrus right	+68 -30 +30	FCSRT free recall	Memory	0.462	0.016
Superior frontal gyrus left	-16 +4 +70	FCSRT free recall	Memory	0.505	0.005
unlabeled*	+20 +32 -30	FCSRT free recall	Memory	-0.502	0.005
Frontal pole right	+46 +48 -16	Letter Fluency (FAS)	Language and executive	0.569	< 0.001
Middle frontal gyrus left	-30 +36 +38	TMTA	Executive	0.393	0.062
Hippocampus left	-22 -10 -18	TMTB – TMTA	Executive	0.446	0.023
First derivative time series as a seed signal					
Posterior middle temporal gyrus right	+60 -26 -12	RCFT immediate free recall	Memory	-0.459	0.018
Vermis 9	+0 -50 -36	RCFT delayed free recall	Memory	-0.396	0.050
Hippocampus right	+32 -8 -22	RCFT copy accuracy score	Visuospatial	0.564	0.001
Precuneus*	+0 -62 +64	JoLO	Visuospatial	0.430	0.030

MNI, Montreal Neurological Institute; FCSRT, Free and Cued Selective Reminding Test; TMTA, Trail Making Test Part A; TMTB, Trail Making Test Part B; RCFT, Rey Complex Figure Test; JoLO, Judgment of Line Orientation.

The associations were evaluated using multiple regression with age, sex, and education as covariates. *P*-values were adjusted for multiple comparisons using false discovery rate correction.

Clusters were named based on the CONN atlas regions of the peak voxels. If the peak voxel was not labeled, the immediately adjacent 26 voxels were scanned, and the cluster was named based on the adjacent voxels' labels, with an asterisk (*) appended to the end of the cluster name. If neither the peak voxel nor the immediately adjacent voxels were labeled in the CONN atlas, the cluster was named as "unlabeled*".

Supplementary Table S5. Correlations of functional networks with neuropsychological performance in healthy older participants

Cluster name	Peak coordinates in MNI space	Neuropsychological assessment	Test domain	*r*-statistic	*P*-value
Pupil diameter time series as a seed signal					
Anterior supramarginal gyrus right	+68 -30 +30	MoCA	Overall cognition	0.605	0.018
unlabeled*	-18 +38 +56	MoCA	Overall cognition	-0.609	0.018
Medial frontal cortex*	-8 +40 -20	FCSRT free recall	Memory	-0.724	0.002
Anterior supramarginal gyrus left	-58 -38 +32	FCSRT free recall	Memory	0.664	0.007
Anterior supramarginal gyrus right	+68 -30 +30	FCSRT free recall	Memory	0.685	0.005
unlabeled*	-18 +38 +56	FCSRT free recall	Memory	-0.732	0.002
Frontal pole left	-10 +64 +0	FCSRT free recall	Memory	-0.636	0.013
Middle frontal gyrus right*	+38 +26 +56	RCFT copy accuracy score	Visuospatial	-0.574	0.034
Anterior cingulate	+0 +32 +24	JoLO	Visuospatial	0.604	0.018
Middle frontal gyrus left	-30 +36 +38	TMTA	Executive	0.621	0.017
First derivative time series as a seed signal					
Vermis 9	+0 -50 -36	RCFT delayed free recall	Memory	-0.613	0.042
Hippocampus right	+32 -8 -22	RCFT copy accuracy score	Visuospatial	0.823	< 0.001
Precuneus*	+0 -62 +64	JoLO	Visuospatial	0.622	0.042

MNI, Montreal Neurological Institute; MoCA, Montreal Cognitive Assessment; FCSRT, Free and Cued Selective Reminding Test; TMTA, Trail Making Test Part A; TMTB, Trail Making Test Part B; RCFT, Rey Complex Figure Test; JoLO, Judgment of Line Orientation.

The associations were evaluated using multiple regression with age and sex as covariates in healthy older participants. *P*-values were adjusted for multiple comparisons using false discovery rate correction.

Clusters were named based on the CONN atlas regions of the peak voxels. If the peak voxel was not labeled, the immediately adjacent 26 voxels were scanned, and the cluster was named based on the adjacent voxels' labels, with an asterisk (*) appended to the end of the cluster name. If neither the peak voxel nor the immediately adjacent voxels were labeled in the CONN atlas, the cluster was named as "unlabeled*".

Supplementary Table S6. Correlations of local correlation with neuropsychological performance

Cluster name	Peak coordinates in MNI space	Neuropsychological assessment	Test domain	*r*-statistic	*P*-value
Pupil diameter time series as a seed signal					
Anterior supramarginal gyrus left	-58 -38 +32	FCSRT free recall	Memory	0.179	0.524
Anterior supramarginal gyrus right	+68 -30 +30	FCSRT free recall	Memory	0.209	0.411
Superior frontal gyrus left	-16 +4 +70	FCSRT free recall	Memory	0.129	0.566
unlabeled*	+20 +32 -30	FCSRT free recall	Memory	-0.061	0.816
Frontal pole right	+46 +48 -16	Letter Fluency (FAS)	Language and executive	-0.175	0.524
Middle frontal gyrus left	-30 +36 +38	TMTA	Executive	-0.063	0.812
Hippocampus left	-22 -10 -18	TMTB – TMTA	Executive	0.335	0.152
First derivative time series as a seed signal					
Posterior middle temporal gyrus right	+60 -26 -12	RCFT immediate free recall	Memory	-0.166	0.652
Vermis 9	+0 -50 -36	RCFT delayed free recall	Memory	-0.191	0.562
Hippocampus right	+32 -8 -22	RCFT copy accuracy score	Visuospatial	-0.280	0.358
Precuneus*	+0 -62 +64	JoLO	Visuospatial	0.051	0.937

MNI, Montreal Neurological Institute; FCSRT, Free and Cued Selective Reminding Test; TMTA, Trail Making Test Part A; TMTB, Trail Making Test Part B; RCFT, Rey Complex Figure Test; JoLO, Judgment of Line Orientation.

The associations were evaluated using multiple regression with age and sex as covariates. *P*-values were adjusted for multiple comparisons using false discovery rate correction.

Clusters were named based on the CONN atlas regions of the peak voxels. If the peak voxel was not labeled, the immediately adjacent 26 voxels were scanned, and the cluster was named based on the adjacent voxels' labels, with an asterisk (*) appended to the end of the cluster name. If neither the peak voxel nor the immediately adjacent voxels were labeled in the CONN atlas, the cluster was named as "unlabeled*".

Supplementary Table S7. Correlations of local correlation with CSF biomarkers

Cluster name	Peak coordinates in MNI space	CSF biomarker	*r*-statistic	*P*-value
Pupil diameter time series as a seed signal				
Frontal pole left	-10 +64 +0	T-tau	-0.204	0.591
Frontal pole left	-10 +64 +0	T-tau/Aβ ratio	-0.163	0.681
First derivative time series as a seed signal				
Frontal pole right	+4 +62 -26	T-tau	0.030	0.979

MNI, Montreal Neurological Institute; CSF, cerebrospinal fluid; T-tau, total tau; Aβ, Amyloid-β 1-42.
The associations were evaluated using multiple regression with age and sex as covariates. *P*-values were adjusted for multiple comparisons using false discovery rate correction.
Clusters were named based on the CONN atlas regions of the peak voxels.
